# Metabolic engineering of *Komagataella phaffii* for the efficient utilization of methanol

**DOI:** 10.1186/s12934-024-02475-1

**Published:** 2024-07-17

**Authors:** Yuanyuan Wang, Ruisi Li, Fengguang Zhao, Shuai Wang, Yaping Zhang, Dexun Fan, Shuangyan Han

**Affiliations:** 1 Guangdong Key Laboratory of Fermentation and Enzyme Engineering, School of Biology and Biological Engineering, South China University of Technology, Guangzhou, China; 2 School of Light Industry and Engineering, South China University of Technology, Guangzhou, China

**Keywords:** *Komagataella Phaffii*, Methanol, Xu5P, Formaldehyde, NAD^+^/NADH

## Abstract

**Background:**

*Komagataella phaffii*, a type of methanotrophic yeast, can use methanol, a favorable non-sugar substrate in eco-friendly bio-manufacturing. The dissimilation pathway in *K. phaffii* leads to the loss of carbon atoms in the form of CO_2_. However, the ΔFLD strain, engineered to lack formaldehyde dehydrogenase—an essential enzyme in the dissimilation pathway—displayed growth defects when exposed to a methanol-containing medium.

**Results:**

Inhibiting the dissimilation pathway triggers an excessive accumulation of formaldehyde and a decline in the intracellular NAD^+^/NADH ratio. Here, we designed dual-enzyme complex with the alcohol oxidase1/dihydroxyacetone synthase1 (Aox1/Das1), enhancing the regeneration of the formaldehyde receptor xylulose-5-phosphate (Xu5P). This strategy mitigated the harmful effects of formaldehyde accumulation and associated toxicity to cells. Concurrently, we elevated the NAD^+^/NADH ratio by overexpressing isocitrate dehydrogenase in the TCA cycle, promoting intracellular redox homeostasis. The OD_600_ of the optimized combination of the above strategies, strain DF02-1, was 4.28 times higher than that of the control strain DF00 (Δ*FLD*, *HIS4*^+^) under 1% methanol. Subsequently, the heterologous expression of methanol oxidase Mox from *Hansenula polymorpha* in strain DF02-1 resulted in the recombinant strain DF02-4, which displayed a growth at an OD_600_ 4.08 times higher than that the control strain DF00 in medium containing 3% methanol.

**Conclusions:**

The reduction of formaldehyde accumulation, the increase of NAD^+^/NADH ratio, and the enhancement of methanol oxidation effectively improved the efficient utilization of a high methanol concentration by strain ΔFLD strain lacking formaldehyde dehydrogenase. The modification strategies implemented in this study collectively serve as a foundational framework for advancing the efficient utilization of methanol in *K. phaffii*.

**Supplementary Information:**

The online version contains supplementary material available at 10.1186/s12934-024-02475-1.

## Introduction

Methanol, an important bulk chemical, is industrially produced from natural gas and various renewable resources through an intermediate syngas [1]. Ongoing research explores the synthesis of methanol from CO_2_, considered a promising avenue for mitigating global warming and achieving global carbon neutrality on a global scale [2]. As a non-food organic C1 feedstock, methanol avoids competition with human food sources and stands out as a carbon substitute for sugar in eco-friendly bio-manufacturing processes [3]. Its appeal lies in both its low cost and abundant sources [4]. Furthermore, owing to its higher degree of reduction compared to the majority of sugars [5], methanol can serve as a primary or supplementary carbon source for the production of reducing chemicals, including alcohols, organic acids, and hydrocarbons, with the expectation of higher yields.

Nature encompasses two main categories of methylotrophic microorganisms: methylotrophic bacteria and methylotrophic yeasts. These organisms possess the natural ability to utilize C1 compounds, such as methanol, as substrates for growth and metabolism [6]. Numerous studies have been conducted on the utilization of methanol by various industrial microorganisms, including *Escherichia coli* [7], *Corynebacterium glutamicum* [8], *Saccharomyces cerevisiae* [9], and *Yarrowia lipolytica* [10]. These investigations involve the introduction and optimization of heterologous methanol assimilation pathways. *Komagataella phaffii* (syn *Pichia pastoris*) [11], a native methanotrophic yeast, is widely used in the industry, utilizing methanol as a carbon source for the production of high value-added products, such as heterologous proteins and biochemicals. This preference is attributed to advantages like strain stability and high cell density fermentation [12, 13]. Despite its widespread use, *K. phaffii* encounters limitations in methanol-based bioindustry due to the inherent toxicity of methanol and its intermediate metabolite formaldehyde to cells, coupled with the loss of carbon atoms in the form of CO_2_ formed through the dissimilation pathway [12]. Addressing these challenges, Cai et al. [14]. overexpressed the endogenous gene *DAS2* in *K. phaffii*, which further drives formaldehyde assimilation, reduces formaldehyde accumulation, and increases biomass fatty acid yield. In general, there is a relative scarcity of studies focused on the efficient methanol utilization in *K. phaffii*.

In a previous study [15], our efforts to mitigate the loss of carbon atoms in the dissimilation pathway of *K. phaffii* involved knocking out the first key enzyme, Fld, in the methanol dissimilation pathway of strain GS115. The resulting dissimilation pathway-blocking strain, ΔFLD, exhibited pronounced growth defects in methanol-containing medium compared to the control strain GS115. Transcriptome analysis indicated that the blocked dissimilation pathway led to the downregulation of the assimilation pathway.

**Fig. 1:**
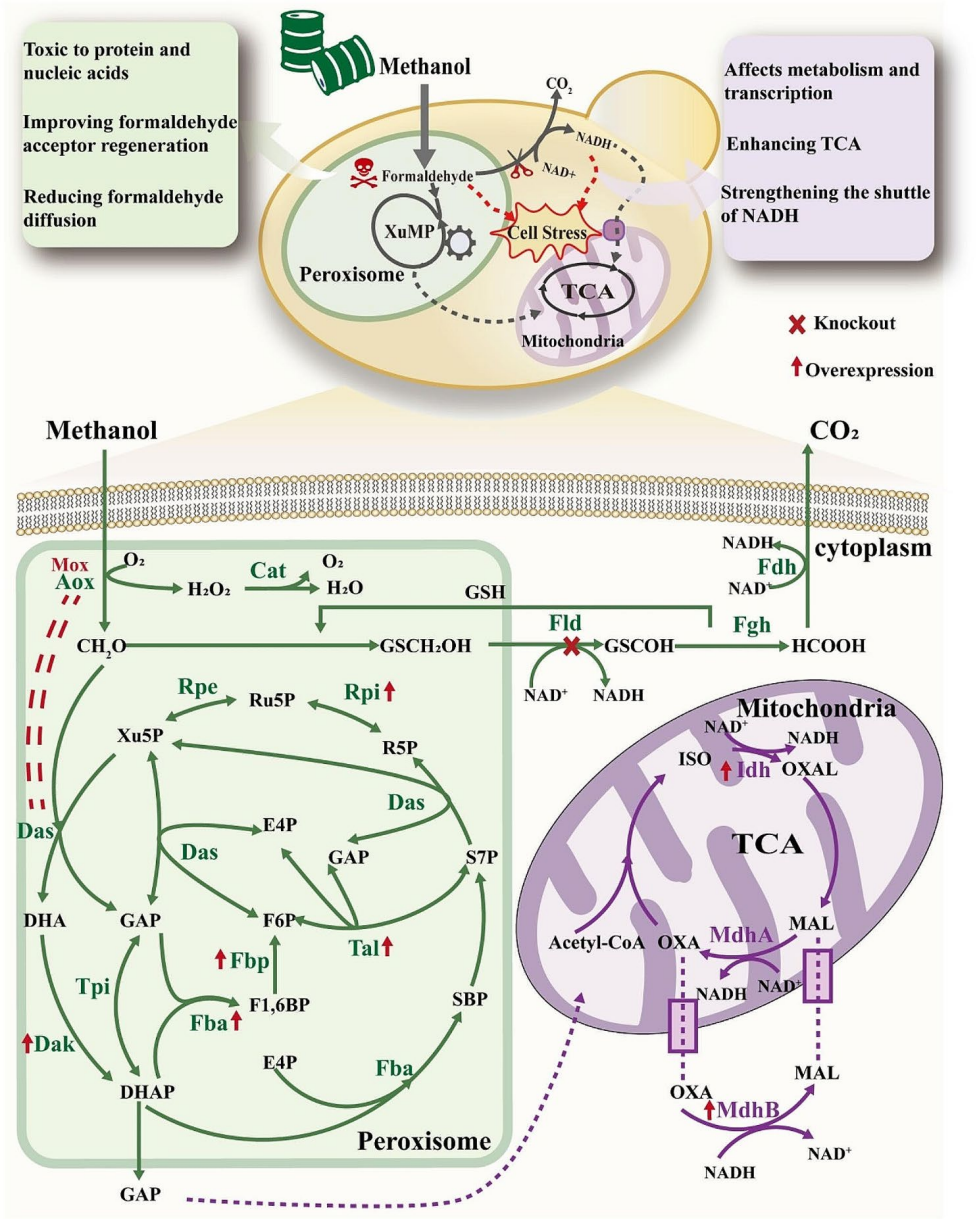
Sketch of methanol metabolic pathway modification in *K. phaffii.* Fld, formaldehyde dehydrogenase; Fdh, formate dehydrogenase; Fgh, S-Formylglutathione Hydrolase; Mox, methanol oxidase from *Hansenula polymorpha* (*Ogataea polymorpha*); Aox, alcohol oxidase; Cat, catalase; Das, dihydroxyacetone synthase; Dak, dihydroxyacetone kinase; Fba fructose-bisphosphate aldolase; Fbp, fructose bisphosphatase; Tpi, triosephosphate isomerase; Tal, transaldolase; Rpi, ribose-5-phosphate isomerase; Rpe ribulose phosphate 3-epimerase; Mdh, malate dehydrogenase; Idh, isocitrate dehydrogenase; GAP, glyceraldehyde 3-phosphate; DHA, dihydroxyacetone; DHAP, dihydroxyacetone phosphate; F1,6BP, fructose-1 6-bisphposphate; F6P, fructose 6 phosphate; E4P, erythrose-4-phosphate; SBP, sedoheptulose 1,7-bisphosphate; S7P, sedoheptulose 7-phosphate; Xu5P, xylulose 5-phosphate; R5P, ribose 5-phosphate; Ru5P, ribulose 5-phosphate; MAL, malate; OXA, oxaloacetate; ISO, isocitrate; OXAL, oxalosuccinate

In this study, we aimed to improve the utilization of methanol in strain ΔFLD through metabolic pathway modification. Our investigation revealed that the growth defect in strain ΔFLD was partially attributed to the excessive accumulation of formaldehyde and a decrease in the NAD^+^/NADH ratio in the presence of methanol. Consequently, we focused on reducing formaldehyde accumulation and increasing the NAD^+^/NADH ratio to improve methanol utilization in strain ΔFLD (Fig. 1). To address the toxicity of formaldehyde, we implemented strategies such as limiting formaldehyde diffusion through the self-assembly of key enzymes Aox1 and Das1 involved in methanol metabolism. Additionally, we promoted formaldehyde assimilation by augmenting the amount of formaldehyde co-reactive substrate, Xu5P. In order to increase the NAD^+^/NADH ratio, NADH production was primarily increased by overexpression of the isocitrate dehydrogenase (Idh) in the TCA cycle. Simultaneously, we facilitated intracellular NADH translocation to mitochondria by increasing the amount of malate dehydrogenase (Mdh) in the malate-aspartate shuttle (also known as malate shuttle) system. Furthermore, the heterologous expression of methanol oxidase (Mox) from *Hansenula polymorpha* was introduced to improve the methanol utilization capacity and growth of the strain in 3% high concentration methanol. In this study, the efficient utilization strategy of methanol provided a valuable base for the application of *K. phaffii* in industrial biotechnology.

## Materials and methods

### Construction of plasmids and strains

The plasmids and strains constructed in this study are listed in Tables 1 and 2.

Strain construction was performed with plasmid-based expression, and plasmids were constructed using the Gibson Assembly Master Mix. All overexpressed genes were performed under the P_*AOX1*_ using PPIC9K or PPICZA as vector backbone, purchased from Invitrogen (Carlsbad, CA, USA). Genes such as *AOX1*, *DAS1*, *FBA* (PAS_chr1-1_0319), *FBP* (PAS_chr3_0868), *TAL* (PAS_chr2-2_0338), *RPIA* (PAS_chr4_0212), *DAK* (PAS_chr3_0841), *MDH* (PAS_chr4_0815), *IDH* (PAS_chr2-1_0120) were amplified by PCR from the genome of *K. phaffii*.

The plasmids were introduced into *K. phaffii* cells by electroporation, using an Eppendorf Eporator (Eppendorf, Germany).

**Table 1:** Plasmids used in this study

Plasmids	Genotype	Source or reference
PPIC9K	Kan^R^	Invitrogen
PPICZA	Zeocin^R^	Invitrogen
PPICKSTD	Kan^R^, P_*AOX1*_-Spytag-Das1-T_*AOX1*_	This study
PPICZCSCA	Zeocin^R^; P_*AOX1*_-Cre-T_*AOX1*_; P_*AOX1*_-Spycather-Aox1-T_*AOX1*_	This study
PHKAM	Kan^R^, P_*AOX1*_-*MDH*-T_*AOX1*_	This study
PHKAI	Kan^R^, P_*AOX1*_-*IDH*-T_*AOX1*_	This study
PHKAP	Kan^R^, P_*AOX1*_-*FBP*-T_*AOX1*_	This study
PHKAA	Kan^R^, P_*AOX1*_- *FBA*-T_*AOX1*_	This study
PHKAD	Kan^R^, P_*AOX1*_- *DAK*-T_*AOX1*_	This study
PHKAT	Kan^R^, P_*AOX1*_-*TAL*-T_*AOX1*_	This study
PHKAR	Kan^R^, P_*AOX1*_-*RPIA*-T_*AOX1*_	This study
PPICZAG	Zeocin^R^, P_*AOX1*_-based expression vector;	This study
PPICZAGMP	Zeocin^R^, P_*AOX1*_-*MDH*-T_*AOX1*_; P_*AOX1*_-*FBP*-T_*AOX1*_	This study
PPICZAGIP	Zeocin^R^, P_*AOX1*_-*IDH*-T_*AOX1*_; P_*AOX1*_-*FBP*-T_*AOX1*;_	This study
PPICZAGMIP	Zeocin^R^, P_*AOX1*_-*MDH*-T_*AOX1*;_ P_*AOX1*_-*IDH*-T_*AOX1*_; P_*AOX1*_-*FBP*-T_*AOX1*_	This study
PPICZAGIPMOX	Zeocin^R^, P_*AOX1*_- *IDH*-T_*AOX1*_; P_*AOX1*_-*FBP*-T_*AOX1*_; P_*AOX1*_-*MOX* -T_*AOX1*_	This study

**Table 2:** Strains used in this study

Strain	Genotype	Source or reference
***E. coli*** TOP 10		
	Wild type	Invitrogen
***K. phaffii***		
GS115	*HIS4* ^*−*^	Invitrogen
ΔFLD	GS115, Δ*FLD*	Lab conserved [15]
DF00	GS115, Δ*FLD*, *HIS4*^*+*^	This study
DF01	GS115, Δ*FLD*, *HIS4*^*+*^, *Spytag-DAS1*	This study
DF02	GS115, Δ*FLD*, *HIS4*^*+*^, *Spytag-DAS1*, *Spycather-AOX1*	This study
DF03	GS115, Δ*FLD*, *HIS4*^*+*^, *FBA*	This study
DF04	GS115, Δ*FLD*, *HIS4*^*+*^, *DAK*	This study
DF05	GS115, Δ*FLD*, *HIS4*^*+*^, *FBP*	This study
DF06	GS115, Δ*FLD*, *HIS4*^*+*^, *RPIA*	This study
DF07	GS115, Δ*FLD*, *HIS4*^*+*^, *TAL*	This study
DF08	GS115, Δ*FLD*, *HIS4*^*+*^, *IDH*	This study
DF09	GS115, Δ*FLD*, *HIS4*^*+*^, *MDH*	This study
DF10	GS115, Δ*FLD*, *HIS4*^*+*^, *MDH*, *IDH*	This study
DF02-1	DF02, *HIS4*^*+*^, *IDH*,* FBP*	This study
DF02-2	DF02, *HIS4*^*+*^, *MDH*,* FBP*	This study
DF02-3	DF02, *HIS4*^*+*^, *MDH*,* IDH*,* FBP*	This study
DF02-4	DF02, *HIS4*^*+*^, *IDH*,* FBP*,* MOX*	This study

### Media and strain cultivation

*Escherichia. coli* Top10 was used as the host strain for the amplification of plasmid. The strains were grown at 37 ℃ and 250 rpm in lysogeny broth (LB) medium (1% [w/v] NaCl, 0.5% [w/v] yeast extract, and 1% [w/v] tryptone; plates containing 2% [w/v] agar), and LBL medium (0.5% [w/v] NaCl, 0.5% [w/v] yeast extract, and 1% [w/v] tryptone).

*K. phaffii* ΔFLD was used as the original strain. The strains were grown in 10 mL yeast extract peptone dextrose (YPD) (1% [w/v] yeast extract, 1% [w/v] peptone, 2% [w/v] glucose) at 30 ℃ and 200 rpm. After the strain was incubated in YPD for 24 h, the fermentation supernatant of YPD was removed by centrifugation at 5000 rpm for 5 min and cells were resuspended with 25 mL of BMMY medium (1% [w/v] yeast extract, 2% [w/v] peptone, 1.34% [w/v] yeast nitrogen base, 100 mM potassium phosphate buffer (PBS) pH 6.0, 1-4% [v/v] methanol) in 250 mL shake flasks with an initial OD_600_ = 1.0. The fermentation was carried out at 30 ℃ and 250 rpm for 2 days.

### Methanol assay

Methanol concentration was determined by high performance liquid chromatography [16]. A 1 mL sample from the fermentation was centrifuged at 6000 rpm for 5 min and the supernatant was filtered through a 0.2 μm filter using a syringe and analyzed on an LC-16 high performance liquid chromatograph (Shimadzu) equipped with a differential refractive index detector (RID-20 A) using a Carbomix H-NP10: 8% column (Sepax Technologies, Inc.). The samples were eluted with 2.5 mM H_2_SO_4_ at a flow rate of 0.6 mL/min at 55 °C for 25 min.

### Formaldehyde assay

Formaldehyde was assayed during growth through the colorimetric Nash assay [17]. 1 mL of sample was centrifuged at 6000 rpm for 5 min. 100 µL of supernatant was pipetted into a 96-well plate (make three replicate wells for each sample), and then 100 µL of Nash reagent [10] (5 M ammonium acetate, 50 mM acetylacetone, and 135 mM acetic acid) was pipetted to mix. The mixture was incubated at 37 °C for 1 h. The absorbance was measured at 412 nm using a microtiter plate reader BioTek Synergy H1 (BioTek). The concentrations were calculated based on a standard curve, freshly prepared with the same batch of the test.

### ROS assay

The cellular ROS level was estimated by using the oxidant sensitive probe 2’,7’-dichlorofluorescin diacetate (DCFH-DA) as described previously [18]. Samples were collected and washed twice with PBS (pH 7.4). The cells were then resuspended in 1 mL 10 mM PBS (pH 7.4) containing 10 µM DCFH-DA, and incubated at 37 °C for 1 h. Fluorescence was measured at λEX 485 nm and λEM 525 nm.

### Measurement of NAD^+^/NADH ratio

The NAD^+^/NADH ratio was assayed using the NAD^+^/NADH Assay Kit with WST-8 from Beyotime (Nantong, China). Following the removal of the medium by centrifugation, 1 mL of pre-cooled lysis buffer was added to the cells, and complete extraction was achieved by breaking the cells with glass beads. Subsequently, the mixtures were centrifuged at 4 °C for 10 min at 12,000 rpm. The supernatant samples were divided into two tubes of 100 µL each and used to measure NADH and total intracellular NAD. Finally, NAD^+^/NADH was measured and calculated according to the manufacturer’s protocol [19]. The protein concentration was measured by the Bradford method [20]. All fluorescence intensity was normalized to the protein level of the supernatant.

### Measurement of propidium iodide (PI) staining

The method of PI staining refers to the previous studies in our laboratory [21]. The cells were collected at 6,000 rpm for 1 min, washed three times in 10 mM phosphate-buffered saline (PBS, pH 7.4). Then, a 10-µL aliquota of 5 mM propidium iodide (PI) was added to 200 µL cell suspension and incubated on a shaker for 30 min at 37 ℃. The cells were washed three times and resuspended in 1.5 mL PBS (pH 7.4). One hundred thousand cells per sample were counted and analyzed by flow cytometry (Beckman Coulter, Fullerton, CA, USA). The data was analyzed by software FlowJo v10.8.1.

### RNA isolation and RT-qPCR

The transcription levels of genes were analyzed by qRT-PCR. Total RNA was extracted from GS115 strains grown in BMMY for 24 h using the hot acid phenol method [22]. For the synthesis of cDNA, PrimeScript™ RT kit from Takara and gDNA Eraser (Perfect Real-time) were used according to the manufacturer’s instructions, and 1 µg of total RNA was used as the template [18]. The mRNA was quantified by qRT-PCR using TB Green^®^ Premix Ex Taq™ (Takara, Japan) [23]. Glyceraldehyde-3-phosphate dehydrogenase gene GAPDH was selected as the house-keeping gene. The primers of RT-qPCR used in this study are listed in Table 2. All experiments were carried out independently in triplicate. The expression ratio of a gene was analyzed by 2^−ΔΔCt^ method [24].

## Results and discussion

### Weak growth of ΔFLD in methanol

**Fig. 2:**
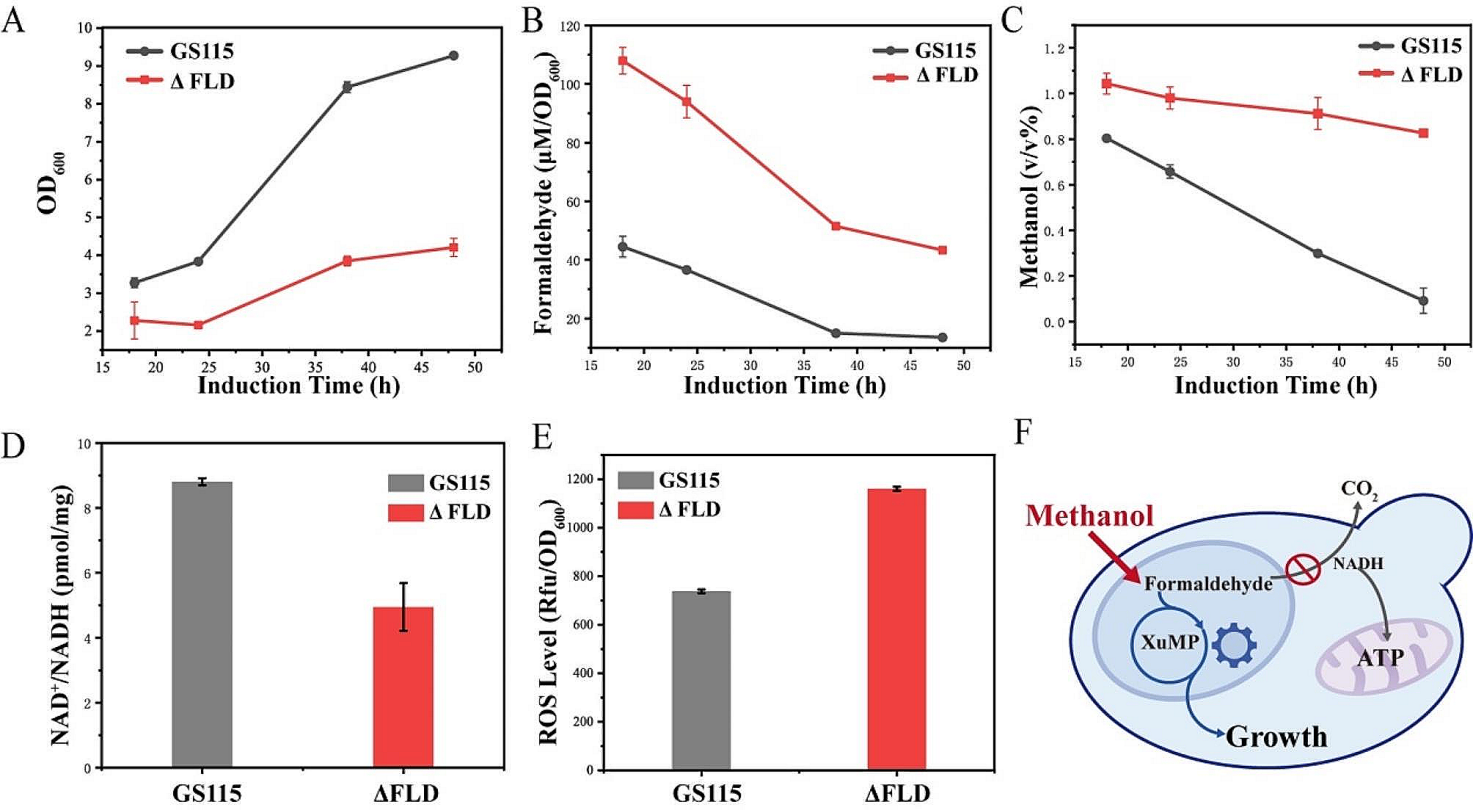
A comparison between strains ΔFLD and GS115 in 25 mL of BMMY medium with 1% methanol. **A** Measurement of growth curve; The x-axes of the culture plots start at 18 h. **B** Measurement of fermentation supernatant formaldehyde; **C** Measurement of fermentation supernatant methanol; **D** Measurement of intracellular NAD^+^/NADH; **E** Measurement of intracellular ROS; **F** Schematic diagram of strain ΔFLD. Error bars represent the standard deviation of 2 or 3 biological replicates

In the dissimilation pathway of methanol metabolism in *K. phaffii*, formaldehyde undergoes initial oxidized by Fld and Fgh to formic acid, subsequently further oxidized to CO_2_ [25]. During the dissimilation of formaldehyde, the conversion of 1 molecule of formaldehyde is coupled with the transformation of 2 molecules of NAD^+^ to NADH. However, excessive dissimilation of methanol leads to a significant loss of C1 substrate in the form of CO_2_, thereby endangering the yield of biomass and target chemicals and reducing the economics of the methanol carbon atom [26]. In the previous research, a strain ΔFLD with a blocked dissimilation pathway was generated [15]. The ΔFLD strain exhibited severe growth defects in medium containing 1% methanol and demonstrated limited methanol utilization (Fig. 2A and C).

Through the experiment, we found that there was a large amount of formaldehyde accumulated in the supernatant of the fermentation broth of the ΔFLD strain, with the accumulated formaldehyde levels being 2 ~ 3 times higher than those in the control strain GS115 (Fig. 2B). Formaldehyde is known to be non-specifically toxic to intracellular DNA and proteins [27]. Strains of *K. phaffii* with compromised integrity face challenges in normal growth in medium where methanol serves as the carbon source, similar to the trend of inhibition of the assimilation pathway due to knockdown of the dissimilation pathway found in previous studies [15]. Measurement of intracellular NAD^+^/NADH, it was found that the intracellular NAD^+^/NADH revealed a lower ratio in the ΔFLD strain compared to GS115 (Fig. 2D). As a cofactor, NAD (NAD^+^ and NADH) participates in over 300 intracellular redox reactions, playing an important role in cellular metabolism [28]. The imbalance in the supply of NAD^+^/NADH caused disrupted intracellular metabolism, affecting the growth of the *K. phaffii* strain in methanol. The methanol dissimilation pathway is a rapid metabolic pathway for the toxic substance formaldehyde and a source of NADH and ATP [29, 30]. Impairment of the dissimilation pathway led to formaldehyde accumulation and intracellular NAD disruption, causing increased intracellular ROS levels (Fig. 2E), which affected methanol metabolism in microbial cell factories. Hence, our studies focused on improving the utilization of methanol by the ΔFLD strain by reducing formaldehyde accumulation and increasing NAD balance.

### Reduction of formaldehyde diffusion by self-assembly of Aox1 and Das1

The excessive accumulation of formaldehyde exerts a toxic effect on proteins and nucleic acids, impeding the efficient utilization of methanol by strains. Therefore, the swift metabolism of formaldehyde is crucial to enhance methanol utilization in strains. Fan [31]et al. fused and expressed Mdh, Hps and Phi in Synthetic methanotrophic *E. coli* by flexible linker (GGGGS)_n_, resulting in improved methanol biotransformation. Thus, preventing the diffusion of toxic intermediates into other intracellular pathways by improving the spatial proximity of enzymes in cascade reactions and the construction of substrate channels through enzyme complexes [32], offering be a promising strategy for enhancing methanol biotransformation [3].

**Fig. 3:**
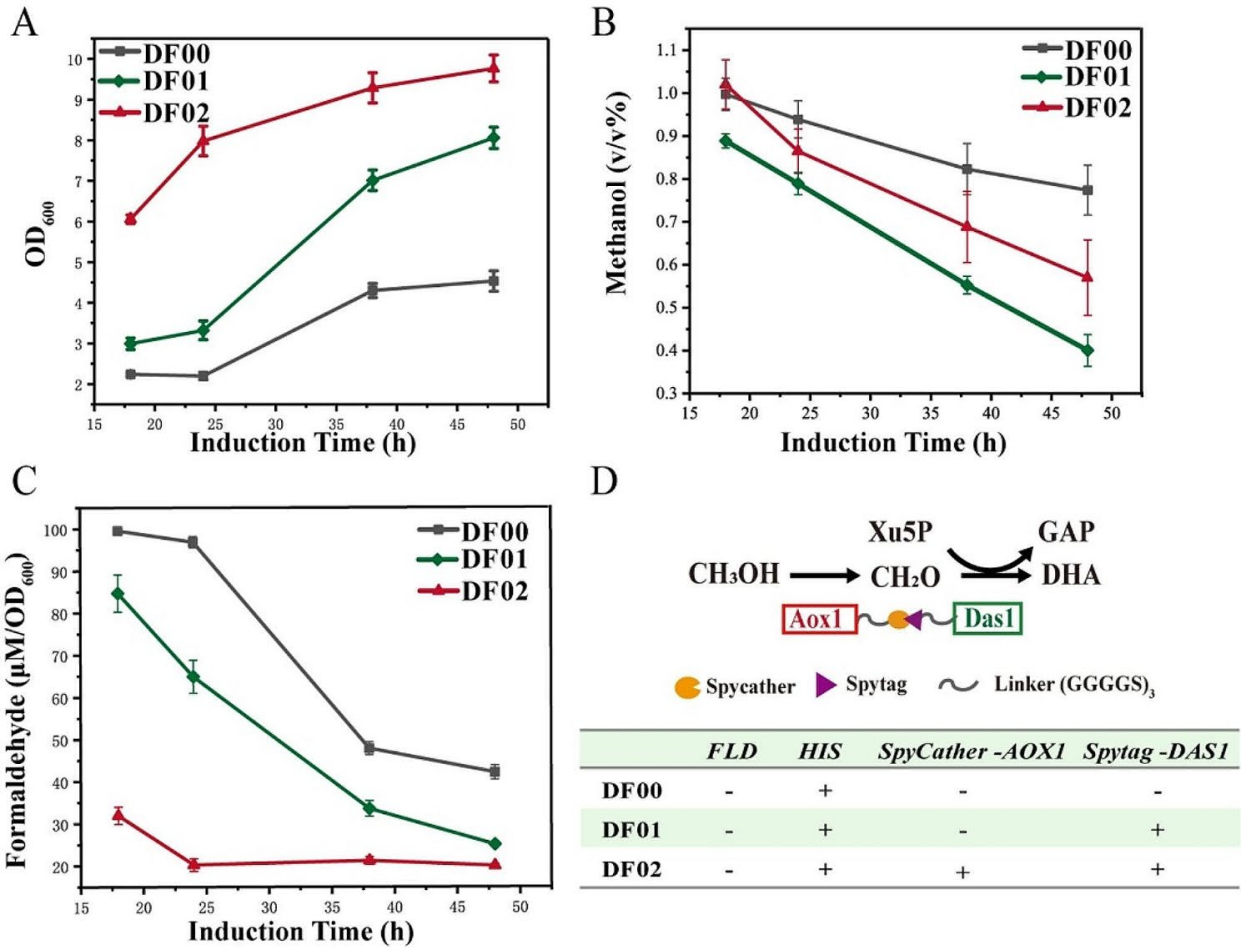
Analysis of strains with the Aox1/Das1 dual enzyme assembly strategy was incubated in 25 mL of BMMY medium containing 1% methanol. **A** Measurement of growth curve; The x-axes of the culture plots start at 18 h. **B** Measurement of fermentation supernatant formaldehyde; **C** Measurement of fermentation supernatant methanol; **D** Schematic diagram of double enzyme assembly. Aox, alcohol oxidase; Das, dihydroxyacetone synthase; Dak, dihydroxyacetone kinase; GAP, glyceraldehyde 3-phosphate; DHA, dihydroxyacetone; Xu5P, xylulose 5-phosphate. Error bars represent the standard deviation of 2 or 3 biological replicates

Methanol is oxidized to formaldehyde by Aox in *K. phaffii*, and formaldehyde is further catalyzed by Das in the carbon metabolism assimilation pathway. Aox and Das are two key enzymes for methanol assimilation. Intracellular assembly of Aox1 and Das1 was performed using the protein scaffold Spytag/Spycather in the strain ΔFLD, with Spycather attached to the C-terminus of Aox1 and Spytag attached to the C-terminus of Das1. Experimental results revealed that, in 1% methanol, the OD_600_ of the recombinant strain DF02 at 48 h was 2.15 times higher than that of DF00 (ΔFLD back-complemented *HIS4*^+^) (Fig. 3A). Notably, assembled strain DF02 exhibited a similar methanol utilization capacity compared to unassembled strains DF00 and DF01 (Fig. 3B). However, DF02 demonstrated superior growth and lower formaldehyde accumulation, with a nearly 52.6% decrease in formaldehyde accumulation in the fermentation supernatant compared to DF00 (Fig. 3C). These results showed that the supramolecular enzyme complex formed by the self-assembly of Aox1 and Das1 helped reducing the diffusion and accumulation of formaldehyde in cells, thereby promoting the growth of the strain in methanol. Moreover, it also indicates that formaldehyde is more toxic than methanol and has a great inhibitory effect on cell growth.

### Promoted formaldehyde assimilation by increased formaldehyde receptor Xu5P

In the process of methanol metabolism, the generated formaldehyde necessitates further metabolized with a co-reaction substrate. The metabolism of formaldehyde is constrained by the availability of the co-reaction substrate, making it imperative to enhance the regeneration of the formaldehyde receptor for efficient methanol assimilation. However, the regeneration of the formaldehyde receptor poses a significant barrier to formaldehyde assimilation. Woolston et al [13] found that activation of the SBPase pathway and reduction of GAPDH in the RuMP cycle significantly enhanced the regeneration of Ru5P.

In the methanol assimilation metabolic pathway of *K. phaffii*, it is necessary for formaldehyde to produce GAP and DHA of Xu5P catalyzed by Das. Consequently, the metabolism of formaldehyde is also limited by the amount of receptor Xu5P. Comparative analysis of transcriptomic data (Table S1) based on ΔFLD strains in glucose or methanol media showed a significant increase in transcript levels of *DAK*,* FBA2*,* FBP1*,* RPIA*, and *TAL2*, key enzymes of the Xu5P regeneration cycle pathway under methanol culture conditions. The intracellular distribution of these enzymes has been analyzed using online protein localization simulations [33], and most of these enzymes are located in peroxisomes (Table S2). This was in contrast to the previously reported notion of an independent set of non-oxidative phosphorylation pathways in peroxisomes to regenerate Xu5P [34].

**Fig. 4:**
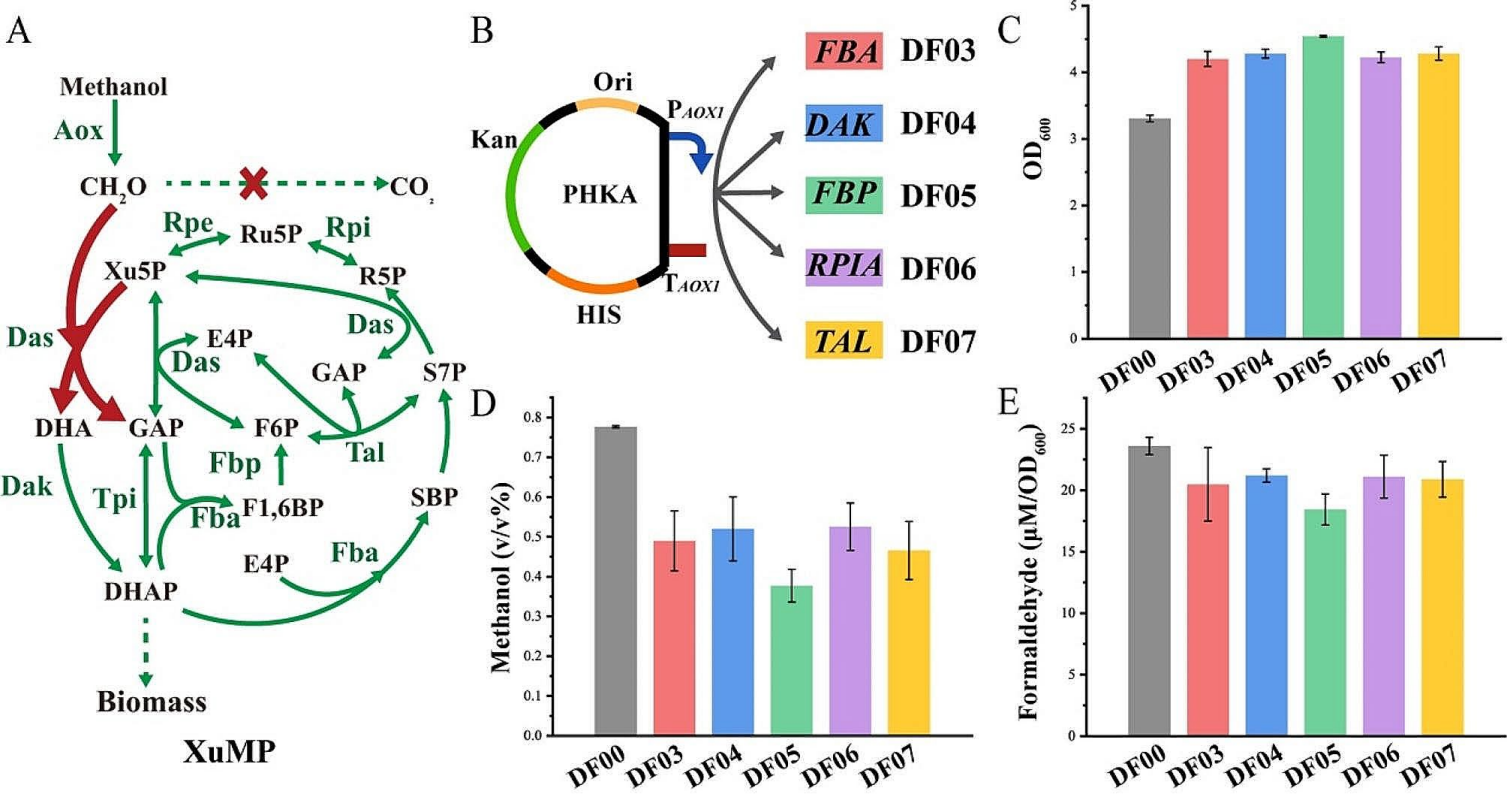
Analysis of strains with overexpression of key enzymes of the XuMP pathway were incubated in 25 mL of BMMY medium containing 1% methanol. **A** Schematic diagram of the methanol assimilation metabolic pathway, Aox, alcohol oxidase; Das, dihydroxyacetone synthase; Dak, dihydroxyacetone kinase; Fba fructose-bisphosphate aldolase; Fbp, fructose bisphosphatase; Tpi, triosephosphate isomerase; Tal, transaldolase; Rpi, ribose-5-phosphate isomerase; Rpe ribulose phosphate 3-epimerase; GAP, glyceraldehyde 3-phosphate; DHA, dihydroxyacetone; DHAP, dihydroxyacetone phosphate; F1,6BP, fructose-1 6-bisphposphate; F6P, fructose 6 phosphate; E4P, erythrose-4-phosphate; SBP, sedoheptulose 1,7-bisphosphate; S7P, sedoheptulose 7-phosphate; Xu5P, xylulose 5-phosphate; R5P, ribose 5-phosphate; Ru5P, ribulose 5-phosphate; **B** Schematic diagram of the overexpression plasmid; **C** Measurement of 48 h growth OD_600_; **D** Measurement of 48 h fermentation supernatant methanol; **E** Measurement of fermentation supernatant formaldehyde. Error bars represent the standard deviation of 2 or 3 biological replicates

Genes for these enzymes were amplified from the *K. phaffii* GS115 genome using PCR technology and overexpressed under the Aox1 methanol-induced promoter in the ΔFLD strain (Fig. 4B). The engineered strains exhibited approximately 30% higher growth compared to the control strain DF00 (Fig. 4C), an increased methanol utilization rate (Fig. 4D), and reduced formaldehyde accumulation in methanol (Fig. 4E). Notably, strain DF05, overexpressing Fbp1, demonstrated a 32% increase in growth at 48 h and a 21.9% reduction in the accumulation of formaldehyde in the supernatant compared to the control strain DF00, showcasing maximal enhancement in formaldehyde receptor regeneration.

### Optimization of intracellular NAD^+^/NADH balance promote strain growth

In methanol-grown methylotrophic yeast, cellular energy is primarily derived through two main pathways: the TCA cycle reaction via the respiratory chain and the methanol dissimilation pathway [35]. Within the dissimilation pathway, each molecule of formaldehyde is accompanied by 2 molecules of NADH production, which then passes H^+^ through the NADH shuttle system to the respiratory chain inside the mitochondria to produce ATP for cell growth [26, 36]. *FLD* knockdown resulted in a reduction in the intracellular NAD^+^/NADH ratio. The NAD^+^/NADH ratio in cells represents the redox state, which is influenced by and in turn regulates metabolic activity, and redox homeostasis is necessary for optimal cellular health throughout the life cycle [37, 38].

**Fig. 5:**
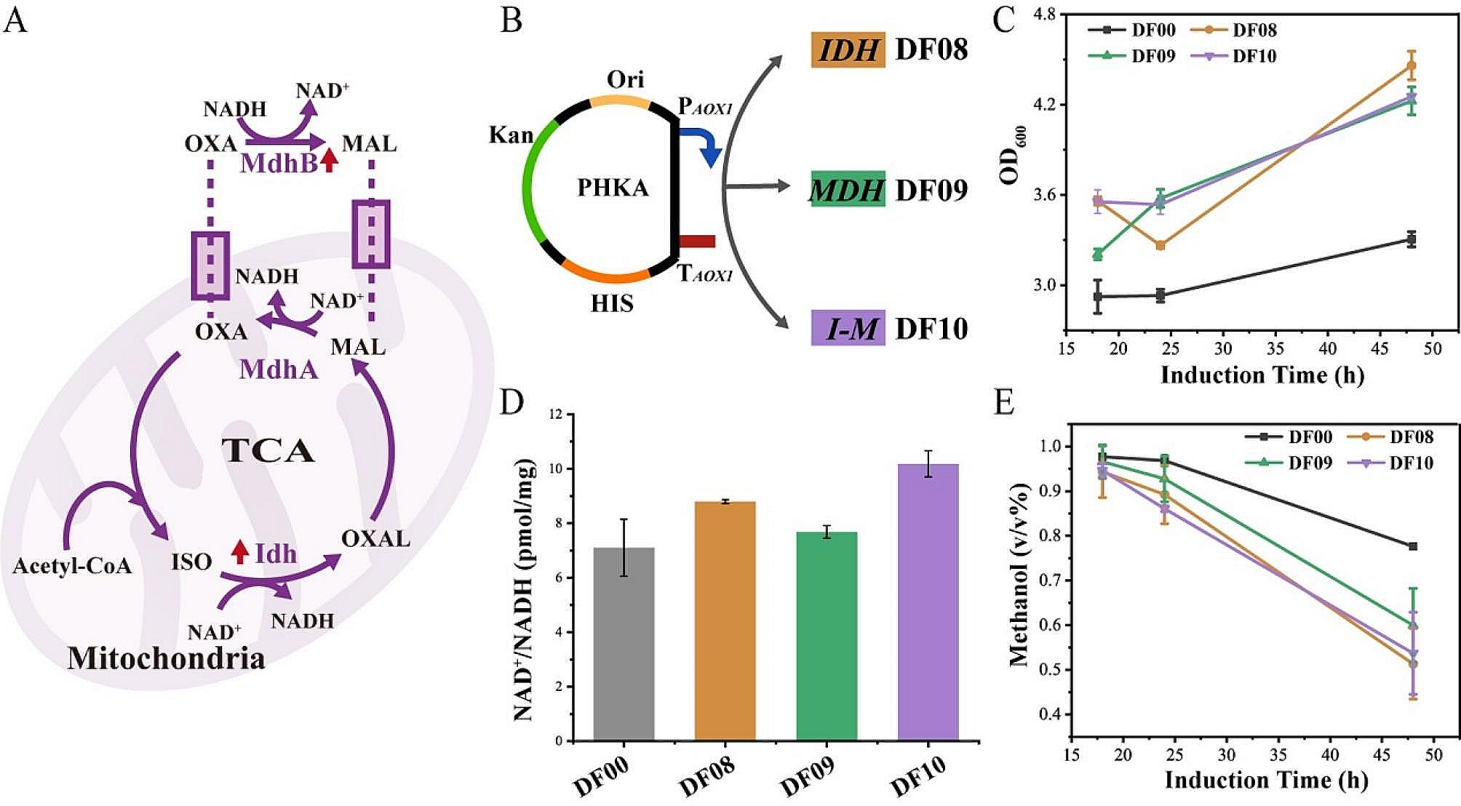
Analysis of strains with enzyme overexpression with NAD^+^ as cofactor was incubated in 25 mL of BMMY medium containing 1% methanol. **A** Schematic diagram of TCA cycle and malate shuttle system, Mdh, malate dehydrogenase; Idh, isocitrate dehydrogenase; MAL, malate; OXA, oxaloacetate; ISO, isocitrate; OXAL, oxalosuccinate; **B** Schematic diagram of expression plasmid; **C** Measurement of growth curve; The x-axes of the culture plots start at 18 h. **D** Measurement of intracellular NAD^+^/NADH; **E** Measurement of methanol in fermentation supernatant. Error bars represent the standard deviation of 2 or 3 biological replicates

Overexpression of Idh in the TCA cycle and Mdh in the NADH malate transport shuttle system was performed individually and in combination in the ΔFLD strain. Experimental results demonstrated improved intracellular NAD^+^/NADH ratio and methanol utilization rates in 1% methanol, leading to a 30% increase in OD_600_ growth. The DF10 strain, expressing Idh and Mdh in combination, exhibited the most substantial increase in NAD^+^/NADH ratio, reaching approximately 10, followed by the DF08 strain expressing Idh (Fig. 5D). However, the growth and methanol utilization capabilities of DF10 strains were not as strong as DF08 strains (Fig. 5C and E), potentially linked to the metabolic stress tendency associated with strains expressing multiple proteins. After 48 h of incubation in 1% methanol, the strain DF08 increased the growth OD_600_ by 34.9% compared with the strain DF00 (Fig. 5C), and methanol residue in the fermentation supernatant decreased by nearly 33.9% ((Fig. 5E). Some studies have observed a decrease in the NAD^+^/NADH ratio during cellular senescence [39, 40]. By enhancing the TCA cycle and NADH shuttle system, the intracellular NAD^+^/NADH ratio can be optimized, improving the intracellular redox state, and promoting methanol utilization in ΔFLD strains.

### Systematic metabolic modification portfolio to improve methanol utilization

**Fig. 6:**
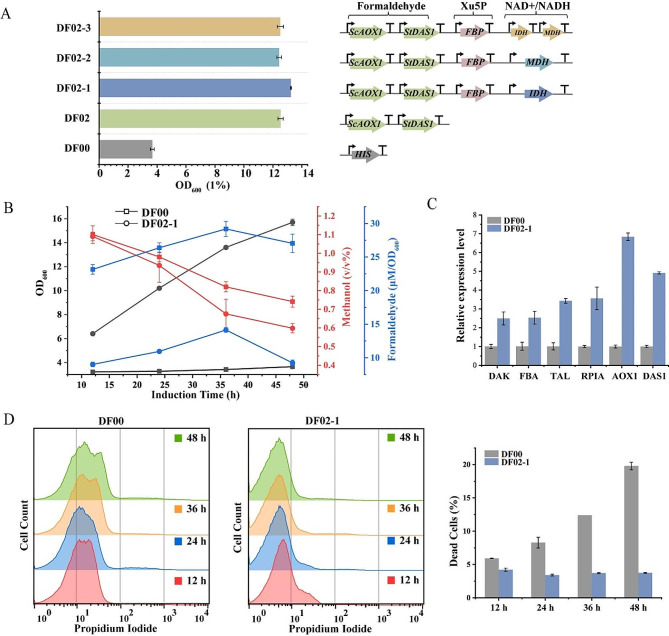
The analysis of strains with an integrated strategy was incubated in 25 mL of BMMY medium containing 1% methanol. **A** Growth OD_600_ of recombinant strains at 48 h; **B** Measurement of OD_600_, methanol content and formaldehyde content of recombinant strains DF02-1 and DF00 grown in 1% methanol; The x-axes of the culture plots start at 12 h. **C** Transcript levels of key enzymes of methanol metabolism; **D** Mortality of recombinant strains in 1% methanol by flow cytometry. Error bars represent the standard deviation of 2 or 3 biological replicates

According to the analysis of the results of the multi-strategy described above, the Aox1/Das1 dual enzyme assembly strain DF02 underwent individual and combined overexpression of *FBP* (XuMP pathway), *IDH* (TCA cycle) and *MDH*. The results, shown in Fig. 6A, revealed that strain DF02-1 exhibited optimal growth performance, with a 4.28-fold increase in OD_600_ over the DF00 control strain after 48 h in 1% methanol. In contrast, strains DF02-2 and DF02-3 displayed lower growth rates compared to DF02. Subsequent comparisons between the final strain DF02-1 and strain DF00 in 1% methanol (Fig. 6B) demonstrated a 20% reduction in methanol residue and a 65.7% decrease in formaldehyde accumulation in DF02-1, accompanied by a 81.1% reduction in cell death compared to DF00 at 48 h (Fig. 6D).

The observed excessive formaldehyde accumulation in the DF00 strain over time, attributed to the *FLD* deletion, led to increased toxicity to cells. This, coupled with a decreased methanol utilization capacity, resulted in elevated methanol levels in the fermentation supernatant, contributing to the dual toxicity of methanol and formaldehyde and an escalating number of dead cells. Transcript levels analysis of key methanol metabolizing enzymes in strains DF00 and DF02-1 in 1% methanol (Fig. 6C) showed that the recombinant strain DF02-1 exhibited 2.5-7 times higher overall transcript levels of methanol metabolizing pathway genes compared to DF00. Notably, the overexpressed *FBP* and *IDH* genes in DF02-1 showed 41.9 and 203.6 times higher transcript levels, respectively (Fig. S3).

In conclusion, the construction of the Aox1/Das1 dual enzyme assembly, along with enhanced Xu5P regeneration and increased intracellular NAD^+^/NADH, successfully promoted methanol assimilation, leading to improved strain growth in methanol.

### Improved utilization of high methanol concentrations in recombinant strain DF02-1

**Fig. 7:**
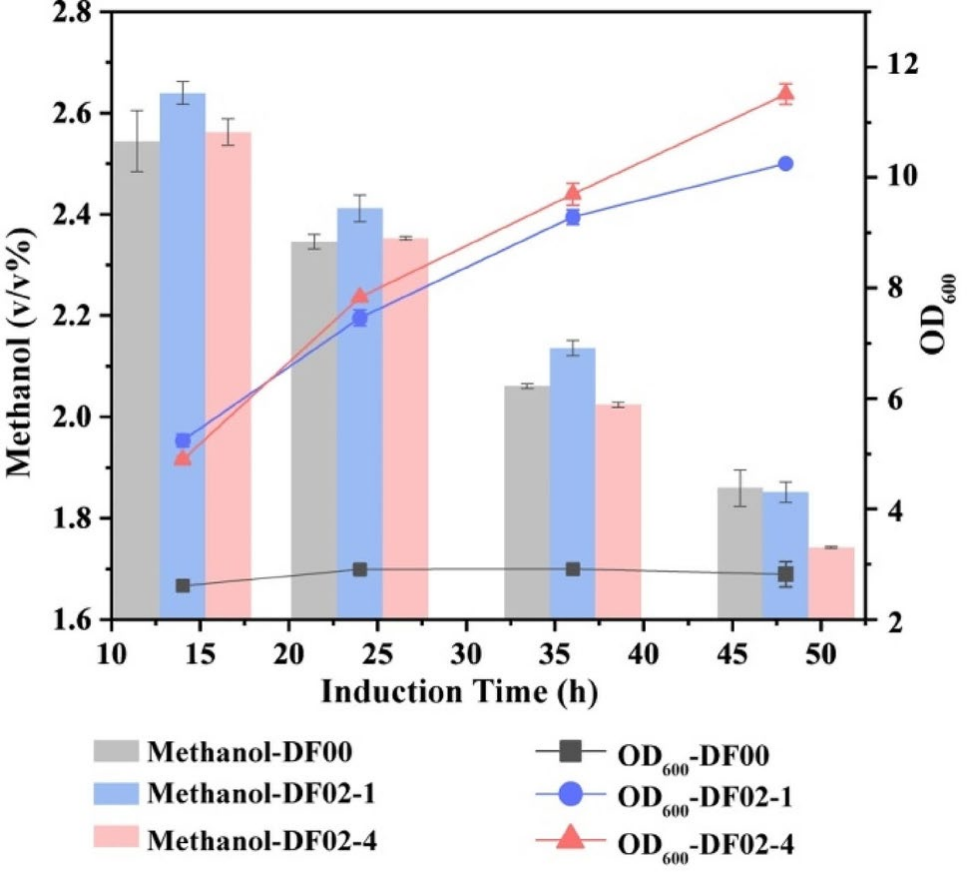
Analysis of recombinant strain DF02-4 in 3% methanol. Error bars represent the standard deviation of 2 or 3 biological replicates. The x-axes of the culture plots start at 14 h

In order to determine the methanol utilization ability of recombinant strain DF02-1 in high concentrations of methanol, we found that the residual amount of methanol in the fermentation supernatant of strain DF02-1 and DF00 were similar in the medium containing 3% methanol, and the methanol utilization ability of the strain was impaired by high concentrations of methanol [35]. In previous studies [41], heterologous expression of *MOX* derived from *Hansenula polymorpha* in *K. phaffii* promoted the methanol utilization in high concentration methanol by recombinant strains. Here we heterologously expressed the enzyme Mox on the basis of the DF02-1 strain, and the designated recombinant strain DF02-4 further improved the utilization of methanol. In BMMY medium containing 3% methanol, the growth OD_600_ of DF02-4 incubated for 48 h was 1.12 times higher than that of DF02-1. At 48 h, the growth OD_600_ of DF02-4 was 4.08 times higher than that of the initial strain DF00, and methanol utilization increased by 10.26%. (Fig. 7). This result indicated that the heterologous expression of *MOX* could effectively improve the growth of the strain in 3% methanol and utilization of methanol.

## Conclusions

In a previous investigation [15], we knocked out the first important enzyme Fld in the dissimilation pathway to reduce the loss of methanol in the form of CO_2_ through the dissimilation pathway of *K. phaffii*. Transcriptomic and metabolomic analyses of the resulting strain ΔFLD revealed a down-regulation in the assimilation pathway, elucidating the growth impairment observed in ΔFLD when cultivated in methanol.

In this study, we analyzed the strain ΔFLD in 1% methanol, revealing subpar methanol utilization, substantial formaldehyde accumulation, and a diminished intracellular NAD^+^/NADH ratio. To address these issues, we employed a multifaceted approach. We assembled Aox1 and Das1 into multifunctional enzyme complexes using the Spytag/Spycather protein scaffold, creating substrate channels to reduce formaldehyde diffusion within the cell. Simultaneously, we augmented the catalytic rate of formaldehyde and enhanced Xu5P regeneration by overexpressing key enzymes in the XuMP pathway, thereby fostering strain growth in methanol and mitigating formaldehyde accumulation. Notably, the NAD^+^/NADH ratio determines the metabolic fluxes of many intracellular pathways and the transcription levels of many genes [28]. As expected, the strategy of overexpressing *IDH* of the TCA cycle and *MDH* of the malate transport shuttle system improved the growth of the strain in methanol and the NAD^+^/NADH ratio. The high concentration methanol weakened the methanol utilization capacity of the strain, nevertheless, the heterologous expression of *MOX* could improve the transformation of methanol by the strain.

Ultimately, by combining multiple strategies—Aox1/Das1 double enzyme assembly, overexpression of Fbp in the XuMP pathway, Idh in the TCA cycle, and heterologous expression of *MOX*—the resulting recombinant strain DF02-4 exhibited a remarkable OD_600_, 4.08 times higher than that of the ΔFLD strain in a medium with 3% methanol. These findings establish a solid research foundation for achieving the economic and efficient utilization of methanol in *K. phaffii*.

### Electronic supplementary material

Below is the link to the electronic supplementary material.

Supplementary Material 1

## Data Availability

No datasets were generated or analysed during the current study.
