# Supplementary material for: Metabolic engineering of Komagataella phaffii for the efficient utilization of methanol
Source: Microb Cell Fact. 2024 Jul 17;23:198. doi: 10.1186/s12934-024-02475-1 (PMC11253385; doi:10.1186/s12934-024-02475-1)
Supplement: Supplementary file 1 — Supplementary Material 1 [file 12934_2024_2475_MOESM1_ESM.docx]

**Supplementary Material**

**Systems metabolic engineering of** ***Komagataella phaffii***

**for the efficient utilization of methanol**

**Yuanyuan Wang^1†^, Ruisi Li^1†^,** **Fengguang Zhao^2^, Shuai Wang^1^, Yaping Zhang^1^, Dexun Fan^1^,**

**Shuangyan Han^1^***

a Guangdong Key Laboratory of Fermentation and Enzyme Engineering, School of Biology and Biological Engineering, South China University of Technology, Guangzhou, China

b School of Light Industry and Engineering, South China University of Technology, Guangzhou, China

†Yuanyuan Wang and Ruisi Li have contributed equally to this work.

*Corresponding author: syhan@scut.edu.cn (Shuangyan Han)

Tel: +86-020-39380618

**Table S1 Transcript levels of the samples**

| Name | Symbol | ΔFLD_D* | ΔFLD_M** | GS115_D* | GS115_M** |
| --- | --- | --- | --- | --- | --- |
| Fba2 | PAS_chr1-1_0319 | 36.44 | 2411.77 | 32.08 | 2664.45 |
| RpiA | PAS_chr4_0212 | 22.04 | 302.16 | 24.85 | 387.97 |
| Tal2 | PAS_chr2-2_0338 | 57.8 | 514.5 | 58 | 531 |
| Dak | PAS_chr3_0841 | 913.96 | 2216.62 | 816.5 | 2537.27 |
| Fbp1 | PAS_chr3_0868 | 426.31 | 2407.09 | 670.72 | 2518.21 |

*in glucose media

**in methanol media

**Table S2 Prediction of protein localization**

| Protein | C-terminal | PTS1 Score | N-terminal | MitoProt Score |
| --- | --- | --- | --- | --- |
| Idh | TSNFTEQVIKNL | -3.212 | MFRQYSRAIRSTPFTRSY | 0.9953 |
| Mdh | NIAKGTAFIAGN | -55.775 | Not predictable | 0.2786 |
| Fba2 | HAAGTFKSESKL | 3.095 | Not predictable | 0.0622 |
| Tal2 | VPSLFRRVLSKL | 7.583 | Not predictable | 0.0074 |
| RpiA | ITSLSVSVPARL | 8.435 | Not predictable | 0.1287 |
| Fbp1 | LTKKIKIQSVNL | -26.614 | not predictable | 0.0841 |
| Dak | ITDAYFKSETKL | -20.743 | Not predictable | 0.072 |

PTS1 predictor version 1: (http://mendel.imp.ac.at/pts1/); MitoProtII program: (https://bio.tools/MITOPROT_II). Scores greater than 0 typically have a predictable PTS1; MitoProtII scores with a probability greater than 0.5 are more likely to be mitochondrial proteins.


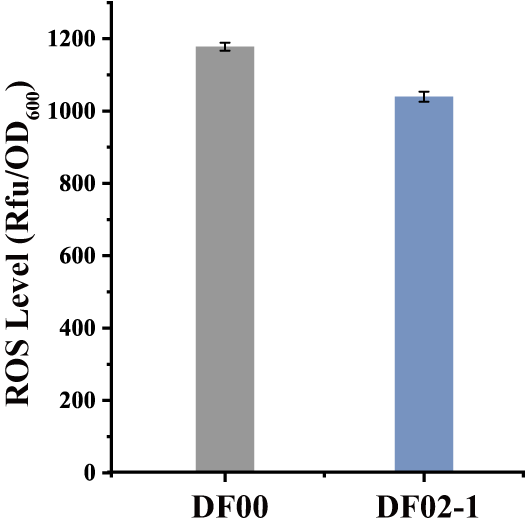


**Figure S1 Analysis of intracellular ROS levels of DF02-1 and DF00 strains in 1% (V/V) methonal at 48h**


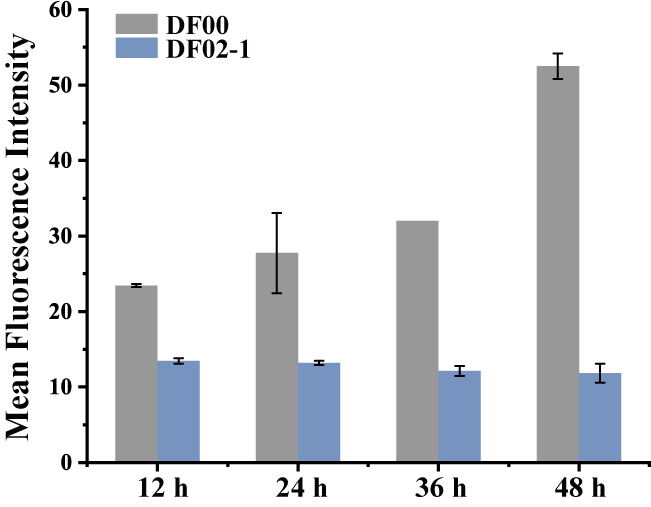


**Figure S2 Mean fluorescence intensity determined by flow cytometry for DF02-1 and DF00**


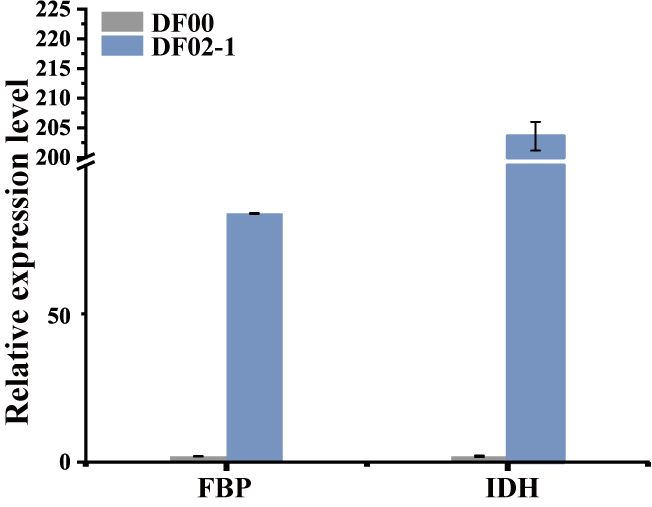


**Figure S3 Transcript levels of IDH and FBP in DF00 and DF02-1**

**
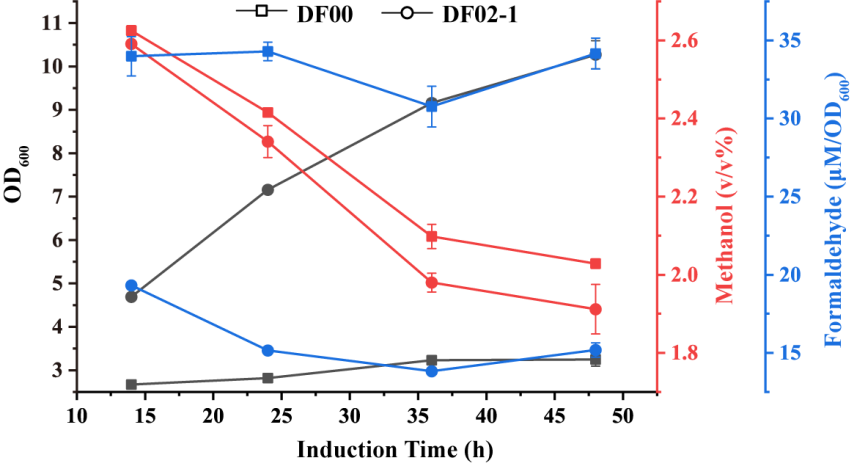
**

**Figure S4 Analysis of strains DF00 and DF02-1 in 3% methanol**
